# Gastrointestinal Bleeding Risk Associated With Pulse Pressure in Patients With Atrial Fibrillation: Retrospective Cohort Study

**DOI:** 10.2196/93347

**Published:** 2026-07-20

**Authors:** Michel Abou Khalil, Carlo El Khoury, Yishi Jia, Maximilian Moersdorf, Christian Massad, Alex El Darzi, Yara Menassa, Mohammad Montaser Atasi, Ghassan Bidaoui, Hadi Younes, Ala Assaf, Mario Mekhael, Eli Tsakiris, Chanho Lim, Charbel Noujaim, Abboud Hassan, Yingshuo Liu, Mary Margot M Maleckar, Rodolfo A Montiel Quintero, Omar Kreidieh, Amitabh C Pandey, Han Feng, Qussay Marashly, Nassir F Marrouche

**Affiliations:** 1 Tulane Research Innovation for Arrhythmia Discovery, Tulane University, 1430 Tulane Avenue, New Orleans, LA, 70112, United States, 1 504-988-3072; 2 Department of Internal Medicine, Emory University, Atlanta, GA, United States; 3 Department of Cardiology, Heart and Vascular Institute, Tulane University School of Medicine, New Orleans, LA, United States; 4 Southeast Louisiana Veterans Health Care System, New Orleans, LA, United States

**Keywords:** atrial fibrillation, pulse pressure, gastrointestinal bleeding, anticoagulation, bleeding risk, arterial stiffness

## Abstract

**Background:**

Patients with atrial fibrillation (AF) face significant bleeding risks, particularly those receiving oral anticoagulation; however, existing risk scores such as HAS-BLED and ORBIT demonstrate limited predictive accuracy. Pulse pressure (PP), calculated as the difference between systolic blood pressure (SBP) and diastolic blood pressure, is a noninvasive marker of arterial stiffness that has been associated with cardiovascular outcomes. However, PP has not been evaluated as a predictor of bleeding in this population.

**Objective:**

This study evaluated whether elevated PP independently predicts major bleeding events, overall and by subtype, in patients with AF after adjusting for established clinical risk factors.

**Methods:**

We conducted a retrospective cohort study using electronic health records from REACHnet, a PCORnet-affiliated clinical data network in Louisiana. A total of 4935 adults (mean age 63.7, SD 11.0 y; n=1606, 32.5% female) with AF between 2010 and 2019 were included via consecutive sampling of all eligible patients. PP was derived from outpatient blood pressure measurements closest to AF diagnosis and analyzed in tertiles (low: <46, middle: 46‐62, high: >62 mm Hg) and continuously per 10 mm Hg. The primary outcome was time to the first bleeding event, a composite of gastrointestinal bleeding, intracranial hemorrhage, and other clinically significant bleeding, identified using *ICD-9*/*ICD-10 *codes. Kaplan-Meier survival curves with log-rank testing were used for univariable analysis. Multivariable Cox proportional hazards regression was adjusted for age, sex, race, comorbidities, medications, and the ORBIT score. A sensitivity analysis applied multivariable logistic regression additionally incorporating SBP. Statistical significance was set at *P*<.05.

**Results:**

Over a 5-year follow-up, 677 out of 4935 (13.7%) patients experienced a bleeding event (intracranial hemorrhage: n=60, 1.2%; gastrointestinal bleeding: n=195, 4.0%; and other bleeding: n=149, 3.0%). Gastrointestinal bleeding differed significantly across PP tertiles (*P=*.007). Kaplan-Meier analysis confirmed lower gastrointestinal bleeding-free survival in the highest tertile (log-rank *P=*.004). No significant differences were observed for intracranial (*P=*.08), other (*P*=.58), or composite bleeding (*P*=.22). In multivariable Cox regression, each 1 mm Hg increase in PP was independently associated with a 1.4% higher gastrointestinal bleeding risk (hazard ratio 1.014, 95% CI 1.001‐1.028; *P=*.04), approximately 15% per 10 mm Hg. This remained significant after adjusting for SBP and ORBIT score (odds ratio 1.013/mm Hg, 95% CI 1.001‐1.025; *P*=.03), while SBP was not independently significant (*P*=.13).

**Conclusions:**

PP independently predicts gastrointestinal bleeding risk in patients with AF beyond established clinical risk factors and validated bleeding risk scores. Unlike prior investigations that examined SBP or diastolic blood pressure components in isolation, this is the first study to identify PP as a predictor of gastrointestinal bleeding in this population. As a readily available, low-cost hemodynamic parameter derived from routine clinical measurements, PP could enhance existing risk stratification tools and inform more personalized bleeding risk management strategies in patients with AF.

## Introduction

Atrial fibrillation (AF) is the most common cardiac arrhythmia and a major contributor to global morbidity and mortality [1,2]. Its prevalence continues to rise, creating growing clinical and economic burdens on health care systems [3]. AF increases the risk of thromboembolic stroke nearly 5-fold, necessitating the use of oral anticoagulation (OAC) as a primary strategy for stroke prevention [4]. However, this therapeutic benefit is offset by a substantial risk of serious bleeding complications, particularly gastrointestinal and intracranial hemorrhages (ICHs), which can lead to hospitalization, premature discontinuation of therapy, and increased mortality [5]. A 2026 meta-analysis of 83 studies (970,248 patients with AF treated with direct oral anticoagulation [DOAC]) confirmed that major bleeding remains a serious and multifactorial complication [6].

To assist clinicians in balancing stroke prevention with bleeding risk, several clinical risk scores have been developed, including HAS-BLED (Hypertension, Abnormal renal and/or liver function, Stroke, Bleeding, Labile INR, Elderly, Drugs and/or alcohol), ORBIT (Older age, Reduced hemoglobin, Bleeding history, Insufficient kidney function, Treatment with antiplatelets), and ATRIA (Anticoagulation and Risk factors in Atrial Fibrillation) [7,8]. These tools incorporate clinical and laboratory parameters such as hypertension, renal dysfunction, age, and prior bleeding history [7]. However, their ability to accurately stratify bleeding risk remains limited. The prospective Murcia Atrial Fibrillation Project-III cohort (2025) demonstrated that all commonly used scores achieved c-indexes below 0.7, with none showing clear superiority, and a 2026 meta-analysis confirmed only modest discrimination for the DOAC score (pooled c-index 0.68) versus HAS-BLED (0.63) [8,9,10]. Therefore, there is a clear need for novel, readily available markers that can enhance bleeding risk stratification.

Pulse pressure (PP), defined as the difference between systolic blood pressure (SBP) and diastolic blood pressure (DBP), serves as a surrogate marker of arterial stiffness and vascular aging [8]. Recent studies have demonstrated its association with adverse cardiovascular outcomes, including stroke, heart failure, and AF [11-13]. Arterial stiffness has also been linked to coagulation imbalance and endothelial injury, suggesting that elevated PP may directly influence hemostatic balance and bleeding susceptibility [14,15].

While previous studies have examined the association of SBP or DBP with bleeding risk in patients with AF, the independent role of PP remains underexplored [16,17]. The F-Create Project confirmed that the incidence of intracranial bleeding in anticoagulated patients with AF increased with higher blood pressure (BP) levels [18]. Critically, the PICASSO (Proximal Internal Carotid Artery Acute Stroke Secondary to Tandem Lesion or Local Occlusion) trial demonstrated that the elevated PP (≥60 mm Hg) was an independent predictor of recurrent hemorrhagic stroke (adjusted HR 6.03, 95% CI 1.04‐34.99) in patients with cerebral microbleeds, suggesting that PP captures a dimension of vascular vulnerability not fully reflected by SBP or DBP alone [15]. To our knowledge, no large-scale study has systematically evaluated the association between PP and major bleeding events, including gastrointestinal, intracranial, and other types, in a contemporary real-world population with AF.

In this study, we aim to evaluate the association between elevated PP and the risk of bleeding in patients with AF, using electronic health record (EHR) data from the REACHnet (Research Action for Health Network). We hypothesize that higher PP, as a marker of arterial stiffness and vascular vulnerability, is an independent predictor of bleeding and may improve risk stratification beyond existing clinical prediction models. If confirmed, PP could serve as a practical, cost-free addition to current bleeding risk assessment tools.

## Methods

### Study Design

This was a retrospective cohort study conducted using EHR data. The study followed patients with AF, examining the association between PP and the risk of major bleeding events. OAC status was recorded as a baseline covariate.

### Setting

The study was conducted using data from REACHnet, a regional PCORnet-affiliated clinical data network based in Louisiana. REACHnet integrates longitudinal clinical data from multiple health systems across the region, capturing information on demographics, diagnoses, medications, vital signs, procedures, and outcomes. The data network draws from a diverse patient population receiving care across outpatient, inpatient, and specialty settings within these affiliated health systems.

The study period spanned from 2010 to 2019. Patients were identified, and their index dates (the date of AF diagnosis) were established within this window. Data collection for baseline covariates occurred prior to the index date, and follow-up continued from the index date until the occurrence of a primary outcome event, loss to follow-up, death, or December 31, 2019, whichever came first.

### Participants

#### Inclusion and Exclusion Criteria

Eligible patients were required to meet all of the following inclusion criteria: (1) a documented diagnosis of AF, defined using *ICD-9* or *ICD-10* (*International Classification of Diseases*) codes, (2) aged ≥18 years at the index date, and (3) available baseline PP data recorded proximal to the index date.

Patients were excluded if they had (1) missing baseline PP data or (2) a documented bleeding event prior to the index date.

#### Participant Characteristics

Baseline characteristics were extracted from EHR data at or preceding the index date and included demographic variables (age, sex, and race/ethnicity), comorbid conditions (hypertension, diabetes mellitus, chronic kidney disease [CKD], congestive heart failure, prior stroke or transient ischemic attack, peripheral artery disease, liver disease, anemia, and thrombocytopenia), and concurrent medication use (OAC type, antiplatelet agents, nonsteroidal anti-inflammatory drugs, beta-blockers, angiotensin-converting enzyme [ACE] inhibitors/angiotensin receptor blockers, calcium channel blockers, and proton pump inhibitors). The ORBIT bleeding risk score was calculated for each patient and incorporated as a composite covariate in adjusted models.

### Sampling Procedures

Patient identification followed a nonprobability, consecutive sampling approach. All patients within the REACHnet who met the eligibility criteria during the study period (2010‐2019) were included in the analysis; no random or purposive sampling was applied. This census-type approach was chosen to maximize sample size and minimize selection bias within the available data source. The index date was defined as the date AF diagnosis had been recorded during the study window.

### Sample Size, Power, and Precision

A total of 4935 patients with AF were identified and included after applying all eligibility criteria. No formal a priori sample size or power calculation was performed. The study enrolled all eligible patients identified within the REACHnet during the study period (2010‐2019). Statistical precision of the primary estimates is reflected in the 95% CIs reported in the *Results* section.

### Measures and Covariates

#### Exposure: PP

The primary exposure was PP, defined as the arithmetic difference between SBP and DBP: PP = SBP – DBP. PP values were derived from outpatient BP measurements recorded in the EHR closest to the index date. For the primary analysis, patients were categorized into tertiles of PP (low, middle, and high) based on the distribution across the full study cohort. In prespecified sensitivity analyses, PP was modeled as a continuous variable, with estimates expressed per 10 mm Hg increment.

#### Primary Outcomes

The primary outcome was the time to the first bleeding event occurring after the index date. Any bleeding was defined as a composite end point comprising (1) gastrointestinal bleeding, (2) ICH, and (3) other clinically significant bleeding. All bleeding events were identified using *ICD-9* and *ICD-10* diagnosis codes recorded in the EHR. Patients were followed from the index date until the first bleeding event, death, loss to follow-up, or the study end date.

#### Secondary Outcomes

Prespecified secondary analyses evaluated the association between PP tertiles and each individual bleeding subtype (gastrointestinal bleeding, ICH, and other clinically significant bleeding) as separate outcomes.

#### Data Sources

All study variables, including the exposure, outcomes, and covariates, were derived exclusively from structured EHR data captured within the REACHnet clinical data network. Diagnoses were ascertained using *ICD-9* and *ICD-10* code mappings applied to the problem list and encounter diagnosis fields. Medication exposures were extracted from prescription records. BP measurements were obtained from vital signs recorded during outpatient encounters. Comorbidity variables were defined using previously validated code sets applied to the longitudinal encounter and diagnosis records.

### Data Analysis

#### Descriptive Statistics

Baseline characteristics were summarized descriptively. Continuous variables were reported as means with SDs or medians with IQRs as appropriate. Categorical variables were reported as frequencies and percentages. Differences in baseline characteristics across PP tertiles were assessed using the Kruskal-Wallis test for continuous variables and chi-square tests for categorical variables. No missing data were identified across the primary exposure, outcome variables, or baseline covariates. Complete case analysis was performed on the full study sample of 4935 patients.

#### Incidence Analysis

Bleeding event frequencies and proportions were summarized for each PP tertile. Kaplan-Meier survival curves were constructed to depict cumulative bleeding-free survival over the follow-up period across tertiles, and differences were assessed using the log-rank test.

#### Multivariable Analysis

Multivariable Cox proportional hazards regression models were used to estimate the association between PP and time to the first bleeding event, adjusting for potential confounders, including age, sex, race, comorbid conditions, concurrent medication use, and the ORBIT bleeding risk score. PP was modeled continuously (per 10 mm Hg increase).

#### Sensitivity Analyses

In sensitivity analyses, a multivariable logistic regression model was additionally fitted, incorporating SBP and the ORBIT score as covariates, to evaluate whether PP retained an independent association with bleeding risk beyond that attributable to SBP alone.

#### Software and Significance Threshold

All statistical analyses were conducted using R (R Development Core Team, version 4.5.1). A 2-sided *P* value of <.05 was considered statistically significant for all tests.

### Ethical Considerations

This study involved a secondary analysis of deidentified EHR data obtained through REACHnet and did not constitute human subjects research requiring institutional review board review under 45 CFR §46.101(b) [19]. Accordingly, institutional review board approval and informed consent were not required. Informed consent for primary data collection was obtained by the participating health systems as part of routine clinical care, and the applicable data use agreements permitted secondary research use without additional patient consent. All data were deidentified prior to analysis in accordance with the HIPAA (Health Insurance Portability and Accountability Act) privacy rule, and no direct patient identifiers were available to the research team at any stage. Data were accessed and stored in a secure, access-controlled environment, consistent with REACHnet governance requirements. No compensation was provided, as no direct participant contact occurred. This paper and all supplementary materials present results in aggregate form only, and no images or descriptions that could identify individual participants are included.

## Results

### Baseline Characteristics

**Figure 1.**
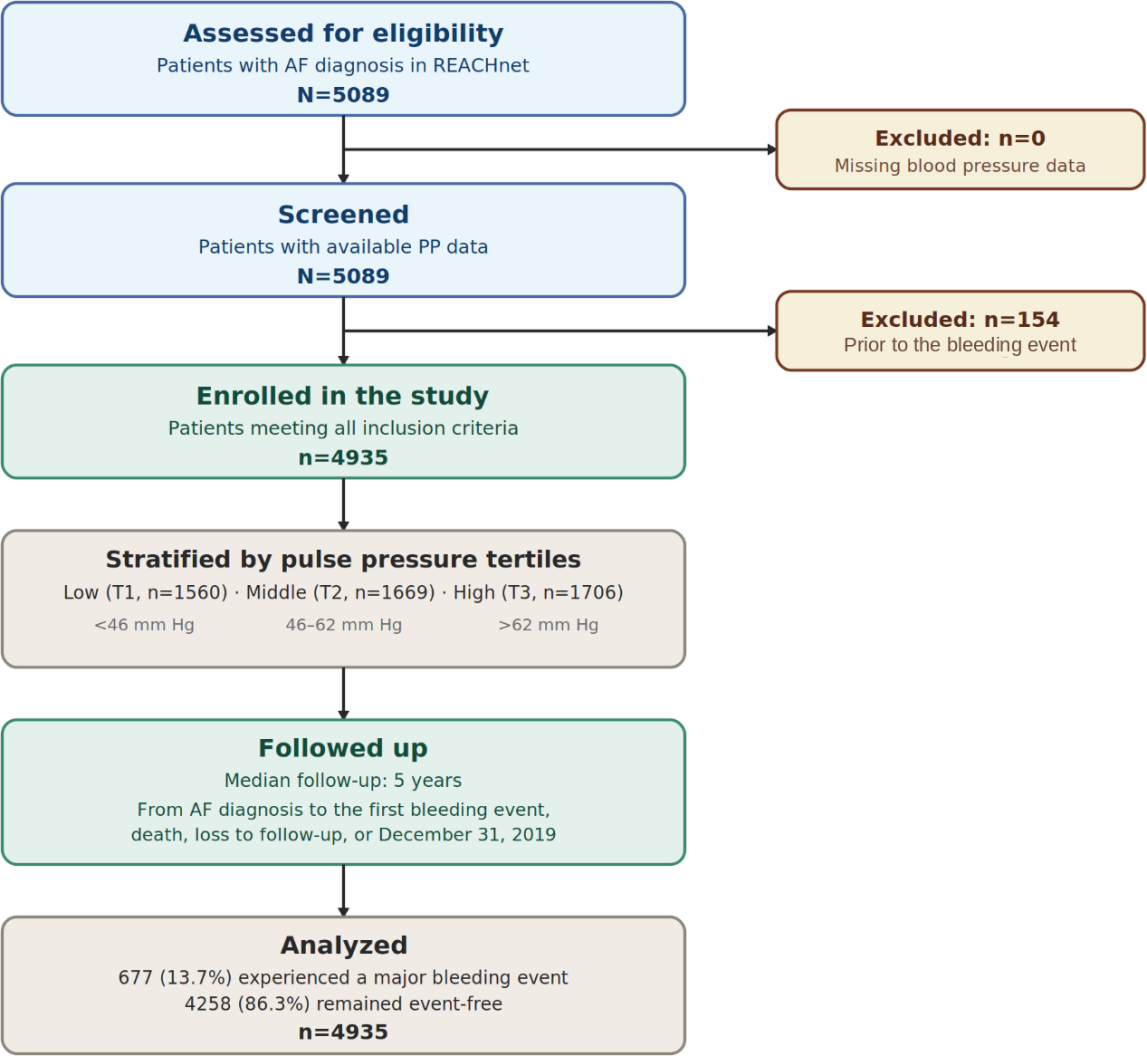
Participant flowchart depicting the selection and follow-up of 4935 adults with atrial fibrillation across each stage of the study, from initial eligibility assessment to final analysis, stratified by pulse pressure tertile (REACHnet clinical data network, Louisiana, United States, 2010‐2019). AF: atrial fibrillation; PP: pulse pressure; REACHnet: Research Action for Health Network.

The participant flowchart is presented in Figure 1. Among 4935 patients with AF, 1560 (n=472, 30.3% female) were in the lowest tertile of PP (T1), 1669 (n=542, 32.5% female) in the middle tertile (T2), and 1706 (n=592, 34.7% female) in the highest tertile (T3).

Patients in the highest PP tertile were significantly older (mean age 66.6, SD 9.3 y) than those in T2 (mean age 63.0, SD 11.2 y) and T1 (mean age 61.3, SD 11.7 y; *P*<.001). Comorbidities, including hypertension, diabetes, CKD, congestive heart failure, peripheral artery disease, anemia, and prior stroke, were more prevalent in the highest PP tertile (all *P*<.05). The use of β-blockers and ACE inhibitors was also more common in higher PP tertiles (Table 1).

**Table 1.** Baseline demographic and clinical characteristics of 4935 adults with atrial fibrillation, stratified by the tertile of pulse pressure (low: <46 mm Hg, middle: 46‐62 mm Hg, high: >62 mm Hg), derived from electronic health records in the REACHnet (Research Action for Health Network) clinical data network (Louisiana, United States, 2010‐2019)^a^.

Characteristic	Low (n=1560)	Middle (n=1669)	High (n=1706)	Total (n=4935)	*P* value
Age (y), mean (SD)	61.3 (11.7)	63.0 (11.2)	66.6 (9.3)	63.7 (11.0)	<.001
Female, n (%)	472 (30.3)	542 (32.5)	592 (34.7)	1606 (32.5)	.03
Hypertension, n (%)	690 (44.2)	803 (48.1)	926 (54.3)	2419 (49.0)	<.001
Diabetes mellitus, n (%)	350 (22.4)	441 (26.4)	598 (35.1)	1389 (28.1)	<.001
Chronic kidney disease, n (%)	244 (15.6)	251 (15.0)	353 (20.7)	848 (17.2)	<.001
Liver disease, n (%)	50 (3.2)	54 (3.2)	53 (3.1)	157 (3.2)	.98
Congestive heart failure, n (%)	402 (25.8)	356 (21.3)	416 (24.4)	1174 (23.8)	.01
Peripheral artery disease, n (%)	268 (17.2)	283 (17.0)	372 (21.8)	923 (18.7)	<.001
Malignant tumor, n (%)	12 (0.8)	17 (1.0)	14 (0.8)	43 (0.9)	.72
Anemia, n (%)	120 (7.7)	107 (6.4)	166 (9.7)	393 (8.0)	.002
Thrombocytopenia, n (%)	7 (0.4)	11 (0.7)	6 (0.4)	24 (0.5)	.43
Stroke, n (%)	152 (9.7)	187 (11.2)	255 (14.9)	594 (12.0)	<.001
Direct oral anticoagulant, n (%)	226 (14.5)	215 (12.9)	242 (14.2)	683 (13.8)	.37
Anticoagulant use, n (%)	553 (35.4)	525 (31.5)	661 (38.7)	1739 (35.2)	<.001
Antiplatelet therapy, n (%)	1 (0.1)	4 (0.2)	5 (0.3)	10 (0.2)	.32
NSAIDs^b^ use, n (%)	266 (17.1)	292 (17.5)	341 (20.0)	899 (18.2)	.06
β-Blocker use, n (%)	228 (14.6)	222 (13.3)	295 (17.3)	745 (15.1)	.004
ACE^c^ inhibitor use, n (%)	270 (17.3)	286 (17.1)	377 (22.1)	933 (18.9)	<.001
Calcium channel blocker use, n (%)	185 (11.9)	164 (9.8)	200 (11.7)	549 (11.1)	.12
Warfarin use, n (%)	134 (8.6)	157 (9.4)	194 (11.4)	485 (9.8)	.02

a Continuous variables are reported as mean (SD); categorical variables as n (%). Group differences were assessed using the Kruskal-Wallis test for continuous variables and chi-square tests for categorical variables.

b NSAIDs: nonsteroidal anti-inflammatory drugs.

c ACE: angiotensin-converting enzyme.

### Bleeding Events and Survival Analysis

During a median follow-up of 5 years, among 4935 patients, 677 (13.7%) experienced a bleeding event, while 4258 (86.3%) remained event-free. Overall, intracranial bleeding occurred in 60 (1.2%) patients, gastrointestinal bleeding in 195 (4.0%), and other bleeding events in 149 (3.0%).

Among the specific bleeding subtypes, only gastrointestinal bleeding showed a statistically significant difference across PP tertiles (*P*=.007), with the highest incidence observed in the upper tertile group. In contrast, rates of intracranial and other bleeding events did not differ significantly between groups (*P*=.08 and *P*=.32, respectively). Full subgroup counts are presented in Table 2.

**Figure 2.**
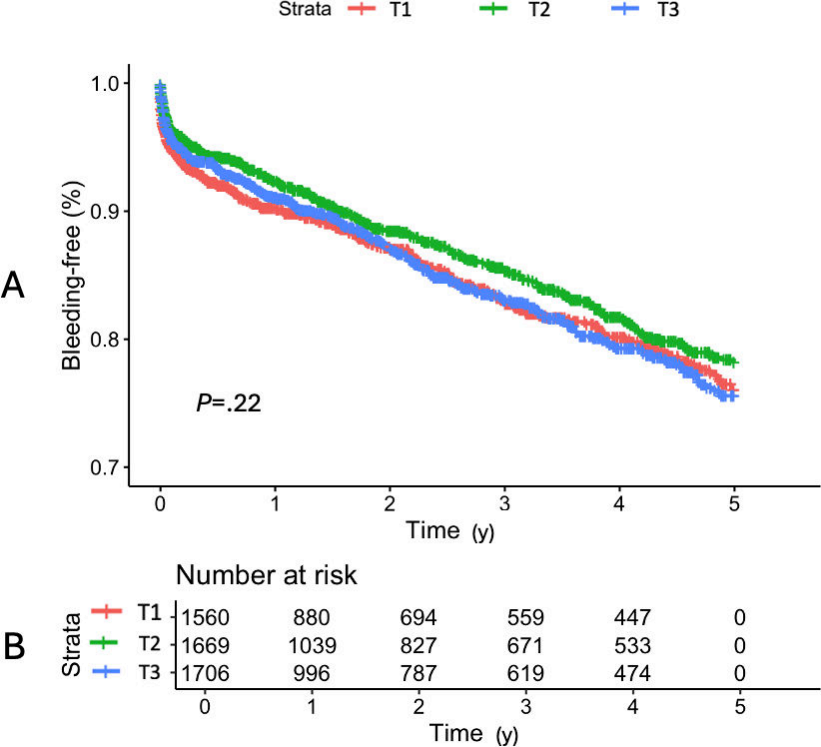
Kaplan-Meier curves depicting composite bleeding-free survival (gastrointestinal bleeding, intracranial hemorrhage, and other clinically significant bleeding) by pulse pressure tertiles (T1: low <46 mm Hg, T2: middle 46‐62 mm Hg, T3: high >62 mm Hg) in 4935 adults with atrial fibrillation (REACHnet [Research Action for Health Network], Louisiana, United States, 2010‐2019); log-rank *P*=.22. (A) Time to any bleeding outcome; (B) number at risk.

**Figure 3.**
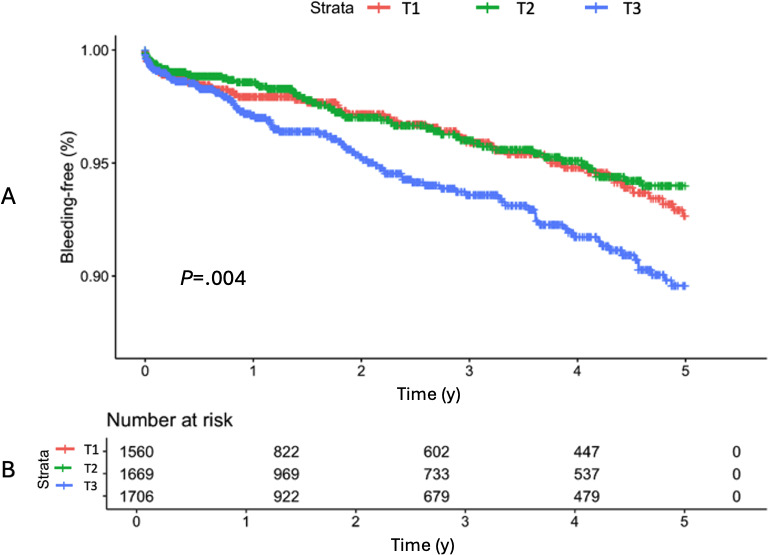
Kaplan-Meier curves depicting gastrointestinal bleeding-free survival by pulse pressure tertiles (T1: low<46 mm Hg, T2: middle 46‐62 mm Hg, T3: high >62 mm Hg) in 4935 adults with atrial fibrillation (REACHnet [Research Action for Health Network], Louisiana, United States, 2010‐2019); log-rank *P*=.004. (A) Time to gastrointestinal bleeding; (B) number at risk.

**Figure 4.**
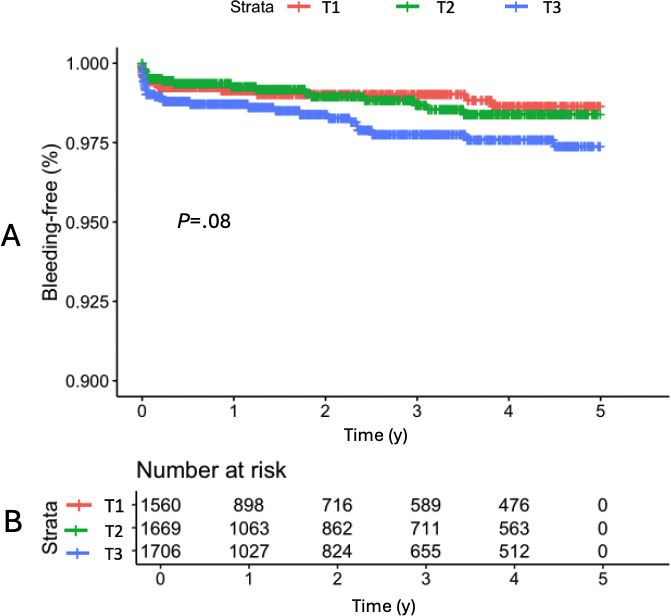
Kaplan-Meier curves depicting intracranial hemorrhage-free survival by pulse pressure tertiles (T1: low<46 mm Hg, T2: middle 46‐62 mm Hg, T3: high >62 mm Hg) in 4935 adults with atrial fibrillation (REACHnet [Research Action for Health Network], Louisiana, United States, 2010‐2019); log-rank *P*=.08. (A) Time to intracranial bleeding; (B) number at risk.

**Figure 5.**
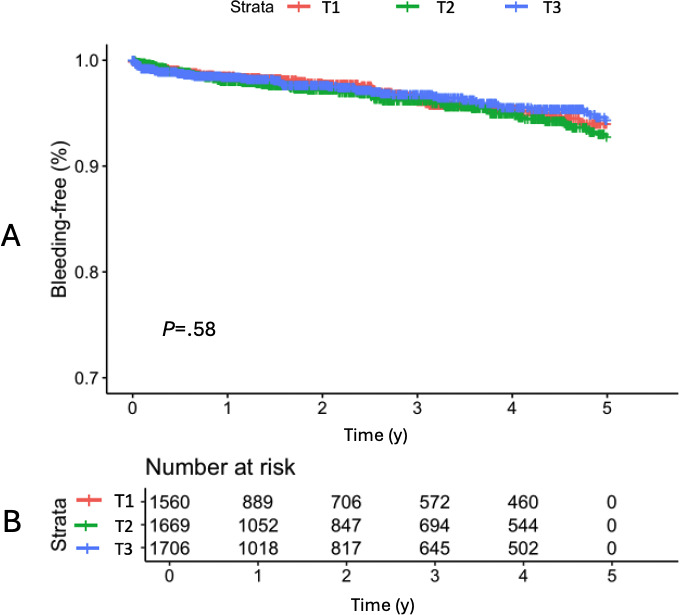
Kaplan-Meier curves depicting other clinically significant bleeding-free survival by pulse pressure tertiles (T1: low <46 mm Hg, T2: middle 46‐62 mm Hg, T3: high >62 mm Hg) in 4935 adults with atrial fibrillation (REACHnet [Research Action for Health Network], Louisiana, United States, 2010‐2019); log-rank *P*=.58. (A) Time to other bleeding; (B) number at risk.

Kaplan-Meier survival analysis demonstrated a statistically significant association between higher PP tertiles and an increased risk of gastrointestinal bleeding (log-rank *P*=.004), with patients in the highest tertile (T3) experiencing the lowest bleeding-free survival over time. In contrast, no significant differences were observed in intracranial bleeding-free survival across tertiles (*P*=.08), nor in the risk of other bleeding events (*P*=.58). For the composite outcome of any bleeding, although some separation of curves was noted, the difference did not reach statistical significance (*P*=.22; Figures 2-5).

In Figures 2-5, Kaplan-Meier curves depicting bleeding-free survival by PP tertile (T1: low <46 mm Hg, T2: middle 46‐62 mm Hg, T3: high >62 mm Hg) in 4935 adults with AF (REACHnet, Louisiana, United States, 2010‐2019). Follow-up extended from the date of AF diagnosis to the first bleeding event, death, loss to follow-up, or December 31, 2019. Outcomes depicted are as follows: Figure 2, composite bleeding (any gastrointestinal, intracranial, or other clinically significant bleeding; log-rank *P*=.22); Figure 3, gastrointestinal bleeding (log-rank *P*=.004); Figure 4, ICH (log-rank *P*=.08); and Figure 5, other clinically significant bleeding (log-rank *P*=.58). Group differences were assessed using the log-rank test.

**Table 2.** Distribution of bleeding events by the pulse pressure tertile in 4935 patients with atrial fibrillation (REACHnet [Research Action for Health Network], Louisiana, United States, 2010‐2019)^a^.

Outcome	Low (<46 mm Hg; n=1560), n (%)	Middle (46‐62 mm Hg; n=1669), n (%)	High (>62 mm Hg; n=1706), n (%)	Total (n=4935), n (%)	*P* value
Intracranial hemorrhage	14 (0.9)	17 (1.0)	29 (1.7)	60 (1.2)	.08
Gastrointestinal bleeding	53 (3.4)	54 (3.2)	88 (5.2)	195 (4.0)	.007
Other bleeding	43 (2.8)	59 (3.5)	47 (2.8)	149 (3.0)	.32
Any bleeding (composite)	218 (14.0)	218 (13.1)	241 (14.1)	677 (13.7)	.22

a Values are presented as n (%). Group differences were assessed using chi-square tests.

### Multivariable Analysis

In multivariable Cox proportional hazards models, PP emerged as a significant independent predictor of gastrointestinal bleeding. Each 10 mm Hg increase in PP was independently associated with a 14.9% higher risk of gastrointestinal bleeding (hazard ratio [HR] 1.149, 95% CI 1.010‐1.318; *P*=.04), even after adjusting for SBP, OAC usage, and a comprehensive set of clinical covariates, including age, sex, race, hypertension, diabetes, CKD, heart failure, anemia, and medication use (HR per 1 mm Hg=1.014, 95% CI 1.001‐1.028; *P*=.04; Table 3). PP remained not significantly associated with intracranial bleeding (HR 1.008, 95% CI 0.986‐1.030; *P*=.50), other bleeding (HR 0.990, 95% CI 0.976‐1.005; *P*=.19), or any bleeding (HR 1.003, 95% CI 0.996‐1.011; *P*=.36).

**Table 3.** Multivariable Cox proportional hazards regression model estimating the association between pulse pressure (per 1 mm Hg increase) and time to the first gastrointestinal bleeding event in 4935 adults with atrial fibrillation (REACHnet [Research Action for Health Network], Louisiana, United States, 2010‐2019)^a^.

Variable	Hazard ratio (95% CI)	*P* value
Pulse pressure	1.014 (1.001-1.028)	.04
Systolic blood pressure	0.994 (0.983-1.005)	.31
Age	1.011 (0.996-1.026)	.15
Sex: female	0.908 (0.666-1.237)	.54
Race	1.109 (0.814-1.512)	.51
Hypertension	2.914 (1.783-4.762)	<.001
Diabetes	0.935 (0.679-1.287)	.68
Chronic kidney disease	1.220 (0.871-1.709)	.25
Congestive heart failure	0.739 (0.523-1.046)	.09
Peripheral artery disease	0.979 (0.685-1.399)	.91
Anemia	1.190 (0.815-1.738)	.37
Thrombocytopenia	0.494 (0.068-3.571)	.49
Stroke	0.927 (0.634-1.355)	.70
Anticoagulation	4.287 (2.733-6.724)	<.001
NSAID^b^	0.603 (0.432-0.841)	.003
β-Blockers	1.493 (1.052-2.118)	.03
ACE^c^ inhibitors	0.717 (0.506-1.014)	.06

a Results are expressed as hazard ratios (HRs) with 95% CI and 2-sided *P* values. In a multivariable logistic regression model adjusting for systolic blood pressure and ORBIT score, pulse pressure (PP) remained a statistically significant predictor of gastrointestinal bleeding (odds ratio 1.013 per mm Hg increase, 95% CI 1.001‐1.025; *P*=.03). In contrast, systolic blood pressure was not significantly associated with bleeding risk (*P*=.13).

b NSAID: nonsteroidal anti-inflammatory drug.

c ACE: angiotensin-converting enzyme.

### Subgroup Analysis by the Anticoagulant Type

**Figure 6.**
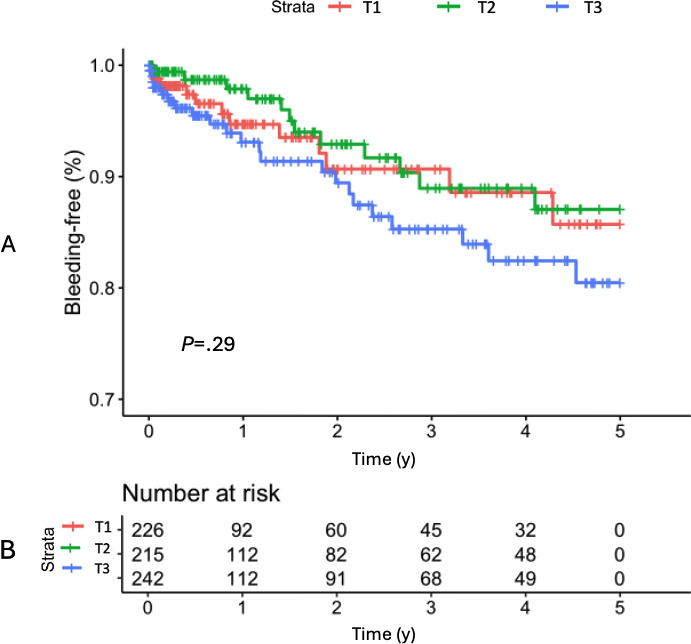
Kaplan-Meier curves depicting gastrointestinal bleeding-free survival by pulse pressure tertiles (T1: low <46 mm Hg, T2: middle 46‐62 mm Hg, T3: high >62 mm Hg) among patients with atrial fibrillation treated with direct oral anticoagulant (DOAC) (T1: n=226, T2: n=215, T3: n=242; REACHnet [Research Action for Health Network], Louisiana, United States, 2010‐2019); log-rank *P*=.29. (A) Time to gastrointestinal bleeding for DOAC; (B) number at risk.

Given the known differential association between the anticoagulant type and gastrointestinal bleeding risk, Kaplan-Meier analyses were performed separately for individuals treated with warfarin and DOAC. Among patients treated with warfarin (T1: n=134, T2: n=157, and T3: n=194), no significant differences in gastrointestinal bleeding-free survival were observed across PP tertiles (log-rank *P*=.96). Similarly, among patients treated with DOAC (T1: n=226, T2: n=215, and T3: n=242), gastrointestinal bleeding-free survival did not differ significantly across tertiles (log-rank *P*=.29; Figures6  7).

**Figure 7.**
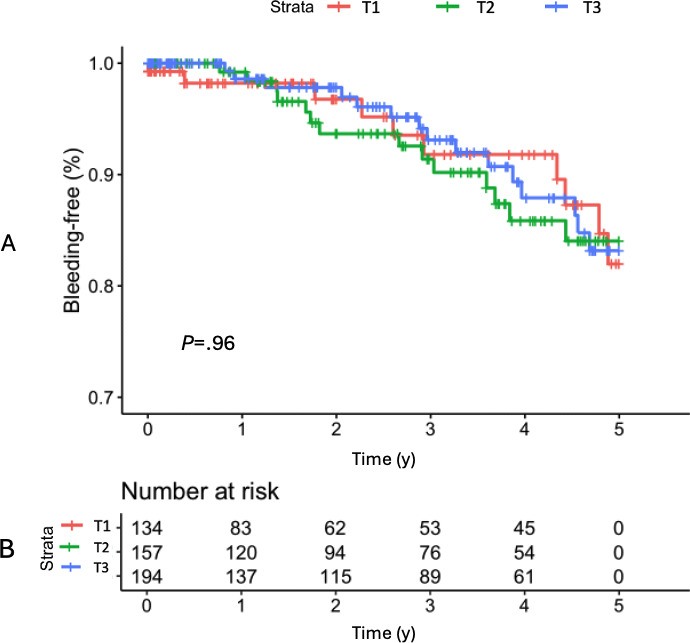
Kaplan-Meier curves depicting gastrointestinal bleeding-free survival by pulse pressure tertiles (T1: low <46 mm Hg, T2: middle 46‐62 mm Hg, T3: high >62 mm Hg) among patients with atrial fibrillation treated with warfarin (T1: n=134, T2: n=157, T3: n=194; REACHnet [Research Action for Health Network], Louisiana, United States, 2010‐2019); log-rank *P*=.96. (A) Time to gastrointestinal bleeding for warfarin; (B) number at risk.

In Figures6  7, Kaplan-Meier curves depict gastrointestinal bleeding-free survival by PP tertiles (T1: low <46 mm Hg, T2: middle 46‐62 mm Hg, T3: high >62 mm Hg), stratified by the anticoagulant type in patients with AF (REACHnet, Louisiana, United States, 2010‐2019). The follow-up extended from the date of AF diagnosis to the first gastrointestinal bleeding event, death, loss to follow-up, or December 31, 2019. Figure 6 depicts patients treated with DOAC (T1: n=134, T2: n=157, and T3: n=194; log-rank *P*=.96). Figure 7 depicts patients treated with warfarin (T1: n=226, T2: n=215, and T3: n=242; log-rank *P*=.29). Group differences were assessed using the log-rank test.

## Discussion

### Principal Findings

This study aimed to evaluate whether elevated PP independently predicts major bleeding events in patients with AF. Consistent with our hypothesis, higher PP was independently associated with an increased risk of gastrointestinal bleeding after adjusting for established clinical risk factors, including SBP and the ORBIT bleeding score. This association was specific to gastrointestinal bleeding and was not observed for ICH, other bleeding subtypes, or the composite bleeding outcome, suggesting a potentially organ-specific hemodynamic mechanism.

### Pulse Pressure and Bleeding Mechanisms

Hypertension is a well-established contributor to bleeding risk in patients with AF. The HAS-BLED score assigns 1 point for SBP >160 mm Hg [20]. High SBP has been linked to intracerebral hemorrhage in patients on warfarin and to higher overall bleeding rates in older patients with AF cohorts [21]. In a recent analysis of Japanese octogenarians with AF, those with poorly controlled home SBP (≥145 mm Hg) had a significantly higher incidence of major bleeding and stroke than those with SBP <125 mm Hg [21]. However, these conventional approaches treat BP as a static or binary variable and do not account for the pulsatile hemodynamic forces that may independently contribute to vascular injury [22]. PP, defined as the difference between SBP and DBP, reflects arterial stiffness and the magnitude of pulsatile stress transmitted to end-organ vasculature, a dimension of hemodynamic risk that existing bleeding scores do not capture [23].

To our knowledge, no prior large-scale study has examined PP in relation to bleeding outcomes in patients with AF. Previous investigations of PP have focused predominantly on its role as a predictor of cardiovascular events such as myocardial infarction, stroke, and cardiovascular mortality, particularly in older adults and hypertensive populations [24]. The extension of PP as a risk marker to bleeding outcomes represents a novel application of this readily available hemodynamic parameter, and this study was designed to address this gap in the literature.

In this large cohort of 4935 patients with AF, higher PP was independently associated with an increased risk of gastrointestinal bleeding. Patients in the highest PP tertile were older and had a higher burden of vascular comorbidities. In multivariable Cox regression, a clinically meaningful and statistically robust increase in gastrointestinal bleeding hazard was observed per 10 mm Hg increment in PP, even after adjusting for SBP, OAC usage, and the ORBIT bleeding score in logistic regression. The consistent direction and statistical significance of this association across both Cox and logistic regression models, with CIs excluding the null in both analyses, strengthen confidence in PP as an independent predictor of gastrointestinal bleeding. The magnitude of effect is clinically plausible, given the hemodynamic burden that elevated PP imposes on the gastrointestinal vasculature. To our knowledge, this is the first study to demonstrate PP as an independent predictor of gastrointestinal bleeding in patients with AF.

Gastrointestinal bleeding remains one of the most common and clinically impactful bleeding complications among patients with AF [25,26]. Among older patients with AF, the incidence of gastrointestinal bleeding was 1.92 per 100 person-years, highlighting the real-world burden and recurrence potential of this outcome [27]. A recent large international study (INTERBLEED) identified age as the strongest predictor of gastrointestinal bleeding in patients with cardiovascular disease, with those aged ≥71 years having more than 4-fold higher risk than those ≤60 years (odds ratio 4.16, *P*<.001) [28]. While aging remains a robust risk factor, its effects are likely mediated through vascular stiffening and elevated PP, a hemodynamic consequence of reduced arterial compliance. In our analysis, PP remained independently associated with gastrointestinal bleeding even after adjusting for age, suggesting that PP may reflect cumulative subclinical vascular damage beyond chronological aging. Mechanistically, PP is a surrogate for arterial stiffness; as arteries stiffen with age and hypertension, they lose elasticity and the ability to dampen the pulsatile output of the heart [29]. This loss of arterial buffering capacity results in the greater transmission of pulsatile energy to downstream microvascular beds, including those of the gastrointestinal tract [23]. The splanchnic circulation, which receives a substantial proportion of cardiac output, may be particularly vulnerable to this augmented pulsatile stress [30].

Our finding that PP was not significantly related to ICH risk hints at possible organ-specific differences. Intracerebral microvessels, which are also sensitive to hypertension, might be more acutely affected by absolute BP spikes or long-term hypertensive remodeling rather than by pulsatile pressure per se [31]. The cerebral vasculature possesses unique autoregulatory mechanisms, including the myogenic response and neurovascular coupling, which actively modulate blood flow across a range of perfusion pressures [32]. These protective mechanisms may partially attenuate the impact of pulsatile stress on cerebral microvessels, whereas the gastrointestinal vasculature lacks comparably robust autoregulatory defenses [33]. In contrast, gastrointestinal bleeding sources (such as submucosal arterioles or angiodysplastic vessels) may be more directly influenced by the ongoing pulsatile stress that PP represents [34]. This pathophysiologic distinction warrants further investigation, but it aligns with our results showing PP’s impact on gastrointestinal bleeds and the relative lack of effect on ICH.

### Clinical Implications

Our findings have several practical implications. Bleeding risk scores such as HAS-BLED and ORBIT could be enhanced by incorporating PP or related measures of arterial stiffness. Current scoring systems typically treat hypertension as a binary variable, without explicitly capturing the hemodynamic burden of vascular stiffness [21]. Given that PP is derived from standard BP measurements already obtained in routine clinical practice, its integration into existing risk models would impose no additional cost or procedural burden [24]. A refined scoring system that incorporates PP as a continuous variable, or as a categorical threshold, could improve the discrimination of patients at the highest risk for gastrointestinal bleeding, thereby enabling more targeted surveillance and intervention strategies.

In our analysis, PP remained an independent predictor of gastrointestinal bleeding even after accounting for SBP and overall bleeding risk as captured by the ORBIT score, whereas SBP alone did not demonstrate a significant association. This underscores that the pulsatile component of BP, rather than absolute systolic values, may better reflect vascular fragility. Since PP can be easily calculated from routine BP readings, its incorporation into clinical workflows may enhance bleeding-risk discrimination, particularly for gastrointestinal events. Clinically, patients with elevated PP might warrant closer surveillance and more stringent BP control. Specifically, clinicians managing patients with AF and elevated PP could consider more frequent hemoglobin monitoring, lower thresholds for endoscopic evaluation of gastrointestinal symptoms, and optimization of antihypertensive regimens with agents known to reduce arterial stiffness, such as ACE inhibitors, angiotensin receptor blockers, or calcium channel blockers, which may preferentially lower PP relative to other drug classes [35,36]. Whether pharmacologic reduction of PP translates into a measurable decrease in gastrointestinal bleeding events remains an important question for future interventional studies [37].

Another forward-looking implication involves the potential role of technology in monitoring PP and arterial health. The proliferation of wearable BP devices enables near-continuous tracking of both systolic SBP and DBP in real-world settings [38]. Frequent out-of-office BP measurements using smartwatch sensors can detect patterns such as episodic hypertension or increased PP with much greater resolution than periodic clinic visits [39]. Integrating these technologies with clinical risk models could facilitate real-time, individualized bleeding-risk assessment. For example, machine-learning algorithms applied to continuous PP data streams could identify patients whose PP trajectories are trending upward, triggering clinical alerts before a bleeding event occurs [40]. Collectively, these approaches highlight the promise of PP as an accessible biomarker bridging traditional risk stratification and digital precision medicine.

### Limitations

This study has several limitations that should be considered when interpreting the findings. PP was derived from a single peripheral measurement, which may not fully capture the dynamic or central hemodynamic burden. However, this proof-of-concept analysis supports the need for future studies employing continuous monitoring or central PP estimation. Second, the retrospective observational design inherently carries risks of residual confounding and bias, despite multivariable adjustment. Prospective validation in diverse cohorts is warranted. Third, the quality of anticoagulation in patients treated with warfarin, specifically prothrombin time–international normalized ratio and time in therapeutic range, was not available in the EHR data and could not be accounted for in the analysis. As anticoagulation intensity is a known determinant of bleeding risk, this represents a potential source of residual confounding that may have influenced the observed bleeding outcomes.

### Conclusion

Our study identifies elevated PP as a significant and independent predictor of gastrointestinal bleeding among patients with AF, representing the first evidence of this association in this population. The organ-specific nature of this finding, with PP predicting gastrointestinal but not intracranial or overall bleeding, suggests that distinct hemodynamic pathways underlie different bleeding subtypes, and that subtype-specific risk modeling may be more informative than composite bleeding end points alone. Unlike existing risk scores that treat hypertension as a binary variable, PP captures the pulsatile hemodynamic burden of arterial stiffness and may therefore improve bleeding risk discrimination when incorporated into clinical prediction models such as HAS-BLED or ORBIT. In our sensitivity analysis, PP remained independently predictive of gastrointestinal bleeding after adjusting for SBP, which itself was not significant, underscoring that the pulsatile rather than the absolute pressure component drives this risk. Patients with AF and elevated PP may represent a high-risk subgroup warranting heightened gastrointestinal surveillance, proactive gastroprotection, and careful selection of anticoagulant agents with favorable gastrointestinal safety profiles. Future research should aim to validate these findings in broader settings and explore how best to incorporate PP into clinical practice, whether by refining risk scores, targeting arterial stiffness reduction pharmacologically, guiding BP targets, or leveraging wearable technology for continuous vascular monitoring.

## Abbreviations

ACE: angiotensin-converting enzyme
AF: atrial fibrillation
ATRIA: Anticoagulation and Risk Factors in Atrial Fibrillation
BP: blood pressure
CKD: chronic kidney disease
DBP: diastolic blood pressure
DOAC: direct oral anticoagulant
EHR: electronic health record
HAS-BLED: Hypertension, Abnormal renal and/or liver function, Stroke, Bleeding, Labile INR, Elderly, Drugs and/or alcohol
HIPAA: Health Insurance Portability and Accountability Act
HR: hazard ratio
*ICD*: *International Classification of Diseases*
OAC: oral anticoagulation
ORBIT: Older age, Reduced hemoglobin, Bleeding history, Insufficient kidney function, Treatment with antiplatelets
PICASSO: Proximal Internal Carotid Artery Acute Stroke Secondary to Tandem Lesion or Local Occlusion
PP: pulse pressure
REACHnet: Research Action for Health Network
SBP: systolic blood pressure
